# Experiences of everyday racism in Toronto’s health care system: a concept mapping study

**DOI:** 10.1186/s12939-021-01410-9

**Published:** 2021-03-10

**Authors:** Deb Finn Mahabir, Patricia O’Campo, Aisha Lofters, Ketan Shankardass, Christina Salmon, Carles Muntaner

**Affiliations:** 1 Faculty of Nursing, University of Toronto, 155 College Street, Suite 130, Toronto, Ontario M5T 1P8 Canada; 2 MAP Centre for Urban Health Solutions, 30 Bond Street, Toronto, Ontario M5B 1W8 Canada; 3 Women’s College Hospital, 76 Grenville St., Toronto, M5S 1B2 Canada; 4 Department of Health Sciences, Wilfrid Laurier University, 75 University Avenue West, Waterloo, Ontario N2L 3C5 Canada; 5 Knowledge Translation Li Ka Shing Knowledge Institute, St. Michael’s Hospital, 209 Victoria Street, Toronto, ON M5B 1T8 Canada; 6 Dalla Lana School of Public Health, University of Toronto, 155 College St., Toronto, Ontario M5T 3M7 Canada

**Keywords:** Everyday racism, Institutional racism, Policy, Concept mapping, Health care

## Abstract

**Background:**

In Canada, there is longstanding evidence of health inequities for racialized groups. The purpose of this study is to understand the effect of current health care policies and practices on racial/ethnic groups and in particular racialized groups at the level of the individual in Toronto’s health care system.

**Methods:**

This study used a semi-qualitative study design: concept mapping. A purposive sampling strategy was used to recruit participants. Health care users and health care providers from Toronto and the Greater Toronto Area participated in all four concept mapping activities. The sample sizes varied according to the activity. For the rating activity, 41 racialized health care users, 23 non-racialized health care users and 11 health care providers completed this activity. The data analysis was completed using the concept systems software.

**Results:**

Participants generated 35 unique statements of ways in which patients feel disrespect or mistreatment when receiving health care. These statements were grouped into five clusters: ‘Racial/ethnic and class discrimination’, ‘Dehumanizing the patient’, ‘Negligent communication’, ‘Professional misconduct’, and ‘Unequal access to health and health services’. Two distinct conceptual regions were identified: ‘Viewed as inferior’ and ‘Unequal medical access’. From the rating activity, racialized health care users reported ‘race’/ethnic based discrimination or everyday racism as largely contributing to the challenges experienced when receiving health care; statements rated high for action/change include ‘when the health care provider does not complete a proper assessment’, ‘when the patient’s symptoms are ignored or not taken seriously’, ‘and ‘when the health care provider belittles or talks down to the patient’.

**Conclusions:**

Our study identifies how racialized health care users experience everyday racism when receiving health care and this is important to consider in the development of future research and interventions aimed at addressing institutional racism in the health care setting. To support the elimination of institutional racism, anti-racist policies are needed to move beyond cultural competence polices and towards addressing the centrality of unequal power social relations and everyday racism in the health care system.

## Introduction

Despite publicly funded health insurance, there is growing recent evidence from the Canadian Community Health Survey, the largest nationally representative dataset, of health inequities for racialized groups in Canada [1–6] and in Toronto [7]. Most recently, in Toronto and the Greater Toronto Area (GTA) communities, racialized groups are six to seven times more likely to test positive for Covid-19 [8]. However, current health care processes and practices do not ensure their health needs are met [9]. With widening health inequities [10–12], developing an effective policy of action requires a better understanding of the mechanisms through which health inequities are distributed [13]. Yet in Canada, there is paucity of data on racialized health care experiences [14, 15]. Historically, in Canada, ‘race’ /ethnic stratification information was not collected in databases, health registries, or in health care settings (primary health care settings or hospitals). Until recently, of the limited previous research conducted on health inequities in Canada, most studies had relied on proxies for racialized groups (e.g. immigrant status or region of origin) [16].

Racial implicit biases on the part of health care providers has been repeatedly empirically demonstrated in systematic reviews [17–20] and there is increasing evidence of its impact. Perceived racism in the health care setting is strongly related to worse mental health for racialized groups [21]; a review of racism and health service utilization demonstrated that racism is associated with a reduced trust in the health care system and health care providers, a reduced adherence to medical regimens and a delay in health care or not seeking health care altogether [22]. More generally, perceived everyday racism is associated with negative health outcomes in the United States [23] and in Canada [24].

Over the last two decades, research has underscored how mechanisms of racism at the macro and meso level are linked to social inequities in health [25–27]. In addition, landmark empirical work by Essed [28] in *Understanding everyday racism*, demonstrated how racism occurs simultaneously at all three levels of society – the macro, meso, and micro levels. Particularly, this work shows how racial/ethnic based discrimination at the micro level (systemic everyday racism) is linked to the activation of underlying power social relations and is *interconnected* to the meso level or institutional level (health care system, labour market, schools, courts) and to the macro level or socio-political structure (economic, political, ideological).

Researchers have advocated that when examining racism and health, all three levels of racism - structural policies (macro), institutional or hospital policies (meso), and individual experiences (micro) - should be examined together [27, 29–32]. This view of an interconnection between levels of racism makes clear that there is nothing ‘distal’ about structural or institutional racism, for racism is also encountered and embodied *every* day [31]. This approach also supports Bradby’s [33] call for an end to the conceptual ambiguity that currently hinders researchers from hypothesizing about mechanisms that include the micro processes of interactions between patients and health care providers that are *connected* to macro (and meso) policy processes. At present, the use of term the ‘institutional racism’ is often used in research only as a description of inequities and consequently, fails to identify clear mechanisms of racism [33].

In this study, ‘race’/ethnicity is defined as a power-based social relation: “a set of social relations that are a subset of the structure of a social system: a hierarchical relation between White and Non-white populations that produces ill health among Non-whites through economic, political and cultural (ideological) relations” [25]. This definition acknowledges ‘race’/ethnicity as a social construct – as an abbreviation for the numerous economic, political, and ideological processes that have operated over time and which have maintained an oppressive division between people [25, 34].

Everyday racism is understood as ‘race’/ethnic based discriminatory behaviours and practices between individuals and includes acts of omission; these behaviours and practices occur daily and therefore may be seen as normal by the dominant group. Everyday racism is activated by unequal power social relations resulting in the unequal treatment and access to resources or services for racialized groups thus maintaining racial inequality in the system [28, 35, 36].

Although the Canada Health Act [37] aims to provide reasonable access to health care services without financial barriers, not all goods and services are covered by this policy [38]. Canada is viewed as a liberal welfare state [39] meaning that the state provision of welfare is minimal [40]. Additionally, Canada’s response to economic pressures and globalization has further weakened an already underdeveloped liberal welfare state [41]. In Ontario, since the financial crisis in 2008, there has been a deepening of neoliberal polices in health care and an erosion of social programmes [40] including unemployment insurance [42]. Empirically, in general, health care neoliberalism has resulted in austerity health care spending, a rollback of universalism, a rise in payments at the time of use, and a privatization of health care delivery [43].

This study focuses on meso level practice and policy specific to the Canadian health care system: a biomedical model of health care delivery and cultural competence policy. Within medical education and in health research, ‘race’/ ethnicity is conceptualized primarily as a biological construct instead of a social construct [44–47]. This view has resulted in health care practices and research findings that have pathologized racialized groups [25, 27, 44, 48, 49].

In health care, cultural competence is a policy that focuses on a health care provider’s individual behaviour when providing patient care and is the main approach used for addressing individual patient differences in the health care setting. Systematic reviews, however, have repeatedly demonstrated its limited effectiveness in health outcomes and equity [50–53]. For some time, cultural competence has been critiqued for its promotion of stereotypes and biases towards racial/ethnic groups in clinical decision making through its superficial focus on *cultural* rituals, thus reifying culture as the source of the ‘problem’ that health care providers must address instead of the *centrality or existence of racism* [54].

A recent systematic review identified that the contribution of specific federal and institutional regulations, policies, and practices towards maintaining institutional racism is absent within the extant literature [55]. More primary research is now needed to help understand how and why everyday racism occurs in the health care setting by identifying social mechanisms or processes. The purpose of this study is to understand the effect of current health care policies and practices on racial/ethnic groups and in particular racialized groups at the level of the individual in Toronto’s health care system.

## Methods

Concept mapping (CM), a semi-qualitative study design as developed by Trochim [56], was used in this study. This participatory research method is useful for understanding complex phenomena such as the experiences of a target population and strongly supports and incorporates the inclusion of participants in the generation, interpretation, and analyses of the data [57, 58]. Qualitative and quantitative methods are used to create a structured visual representation in the form of maps and graphs which portray how a group of individuals view a particular issue. CM allows for an exploration of multiple themes and a comparison of similarities and differences in opinions within a community [58]. CM has been used in public health to identify Toronto neighbourhood factors that influence intimate partner violence [59], to engage communities as part of a health and human services needs assessment in Toronto [60] and to identify differences in the opinions on barriers to cancer screening among South Asians in Ontario [61].

Participants for this study were recruited from Toronto and GTA communities. As recommended by Kane and Trochim [58], purposive sampling for heterogeneity was used in order to sample for a diversity of perspectives specific to the research focus. Participants were comprised of two main groups: health care users and health care providers. Participant eligibility was group specific. Recruited health care users were participants who had had a negative experience in Toronto or the GTA health care system within the past 5 years, age 16 years or older, and were able to write in English; given that the focus was on the experience of health care users, there was an oversampling for this group. Recruited health care providers were front line providers (e.g. nurse, doctor, social worker, and pharmacist) who had at least 1 year of practice experience working in Toronto or the GTA.

### Concept mapping activities

Data collection activities were approved by the University of Toronto’s Research Ethics Board. Data collection occurred from October 2018 to July 2019. A participatory approach to CM activities was used in this study and are described well by Burke and colleagues [57]. CM consists of four participant activities: brainstorming, sorting, rating, and mapping. To minimize fatigue, participants are not required to complete all CM activities [58]. There were participants that used the ‘opt out’ option after completing one or two activities and therefore, not all participants completed all activities. With the exception of the mapping activity which took place at a meeting room at the Centre for Urban Health Solutions in downtown Toronto, concept mapping activities took place on-line using the Concept System® Global MAX™ [62] software. As recommended by Kane and Trochim [58], a pilot test was completed for each of the CM activities. CM activities and pilot testing specific to this study are explained below.

#### Brainstorming activity

Brainstorming is the process of generating statements. During the brainstorming activity, participants generated statements in response to a focal question. The goal for participants was to generate as many statements in response to the focal prompt based on experiences. The intent in this study is to capture a wide range of mechanisms contributing to disrespect and mistreatment. The question used in this study was “One way in which patients feel disrespected or feel mistreated when seeking good quality health care (service) is…?”. For health care users the intent was to make explicit, based on experience, how disrespect or mistreatment occurs for patients when receiving health care. For health care providers, the intent was to make explicit, based on knowledge, how current processes may result in disrespect or mistreatment for health care users when receiving health care. Table 1 provides the definition for health care and health care services given to participants prior to starting the brainstorming activity.

**Table 1:** Definitional terms provided to participants

Term	Definition
Health care	Health care that is provided in any health care setting: a family doctor’s office, a specialist’s office, a Telemedicine video conference, a walk-in clinic, a community health care centre, or a hospital.
Health care services	Health care services include the health care and/or services provided by support staff (e.g. receptionist, secretary, coordinator) or health care providers (e.g. doctor, nurse, social worker, physiotherapist, pharmacist) in any health care setting.

#### Sorting activity

For the sorting activity, the goal is for each participant to create a unique classification or pile of similar and dissimilar statements [58]. During this activity, the responses or statements generated from the brainstorming activity were sorted individually by participants into conceptually similar piles. To accomplish this, participants were asked to sort or place individual statements into piles that ‘make sense’.

Multidimensional scaling as developed by Anderberg [63] and Everitt [64] was used to represent the participants’ aggregated sort data onto a two-dimensional configuration. Hierarchical cluster analysis was then used to group the participants’ statements into distinct conceptually similar clusters of statements [58]. These statistical analyses were performed using the Concept System® Global MAX™ [62] software. The reliability measurement for CM is the stress value. A good stress value is below 0.36 [56] and indicates a good statistical fit – that the underlying conceptual phenomenon is generally agreed upon by participants [58].

#### Mapping activity

To support conceptual clarity, the aim of the mapping activity is for participants to interpret the responses from the focal prompt [58]. During this activity, participants reviewed statements in each cluster from a draft cluster map in order to confirm that the statements located in each cluster were conceptually similar. Participants then discussed possible final labels for each cluster and also considered possible cluster mergers or separations. All participants agreed on a five-cluster map solution; that each cluster of the five labelled clusters were distinguishable and non-redundant. The researchers then used this labelled five-cluster map to identify major conceptual regions; these regions were used to depict higher level themes specific to the different regions of the cluster map.

#### Rating activity

The rating activity explicitly focuses on the participant’s perceptions or opinions of importance; it is a process whereby participants individually assign their own values to a statement’s importance [58]. Specific to this study, we wanted to get a sense of the participant’s overall perceptions on the importance of each statement in terms of discrimination based on ‘race’/ethnicity as a reason for the challenges experienced when receiving health care. The intent was to identify mechanisms of ‘race’/ethnic based discrimination of everyday racism. This activity, however, does not reflect the frequency or pervasiveness of disrespect or mistreatment or everyday racism in the health care system.

We also used the rating activity to identify areas considered most important for action/change based on racial/ethnic discrimination as determined by racialized health care users; the rating questions used in this study were: ‘Rate how important discrimination based on ‘race’/ethnicity is as a reason for experiencing these challenges’ and ‘Rate how important each statement is for action or change’. To get an understanding of the relative importance of each statement in relation to the other statements, participants used a Likert-type response scale with a range from one to five. Participants used this scale to rank statements from ‘relatively unimportant’ to ‘extremely important’. To compare the qualitative differences and similarities between participants, aggregated cluster averages were divided into three categories: ‘high’ (statements rated 3.8 or higher), ‘moderate’ (statements rated between 3.7 and 2.9), and ‘low’ (statements rated 2.8 or lower).

#### Pilot tests

Prior to completing the brainstorming activity, a pilot test of the focal prompt was completed with a mock group. Based on the empirical literature, given that participants tend to under report personal experiences of discrimination and tend to report more discrimination for their racial/ethnic group [65], the term ‘discrimination’ was excluded from the focal prompt. Also, as recommended [66], since questions explicitly framed about ‘race’/ethnicity have the potential for interviewer effects – whereby participants report the information that they believe the interviewer is interested in receiving – a two-stage approach was used to offset these concerns. Within CM, this two-stage process was achieved through the use of a broad focal prompt that asked about negative health care experiences followed by a rating question specific to racial/ethnic discrimination. To ensure that all statements were sortable and rateable, a pilot test of the sorting and rating activities was completed. For statements that were deemed to be not sortable or rateable, these statements were excluded from the final statement list.

#### Idea synthesis

Ideas synthesis is the process of cleaning the data derived from the brainstorming activity in order to create a final data set or list of unique statements that are relevant, representative, and non-redundant. The goal of the idea synthesis is to generate a manageable data set of statements for the next activities in CM: sorting and rating [58]. The idea synthesis process was completed by the researchers and according to CM guidelines [58]. All statements were corrected for grammar and punctuation, split compound statements, and checked to ensure that they answered the focal prompt.

The final idea synthesis list contained 35 edited, rateable, non-redundant statements. A final total of 35 statements is in keeping with recommendations that the number of statements should not exceed 40 in order to reduce the demands of the participants during the rating activity [67]. Using Excel, an audit trail of the idea synthesis process was recorded (i.e. how statements were merged, edited, or deleted).

### Map generation

The cluster map represents the statements in distinct non-overlapping conceptually similar clusters. Each point or number on the map represents a unique statement from the list of 35 statements. Statements that are closer together on the map may illustrate the degree to which statements are conceptually similar; statements that are conceptually dissimilar are farther apart.

Pattern match graphs compare differences and similarities between cluster rankings. Overall, this graph highlights areas of consensus and difference in rating priorities. A correlation coefficient *r* value of 1.0 indicates complete agreement (depicted as horizontal lines) between variables, whereas an *r* value of − 1.0 indicates that ratings were in complete disagreement (depicted as diagonal lines) [58]. For this study, the go-zone graph selected identifies statements that were highly rated based on two rating questions in order to identify which statements should be acted upon for change.

### Analytic categories

For the ‘race’/ethnic stratification, the analytic categories were coded into racialized and non-racialized groups. The categories comprising racialized groups (in alphabetical order) were: Arab, Black, Chinese, Filipino, Japanese, Korean, Latin American, South Asian, Southeast Asian, West Asian, White, and other. The non-racialized group consisted of all participants who self-identified as White. These analytic categories are used by Statistics Canada and therefore selected for this study in order to situate findings with previous research on racism in Toronto and Canada.

## Results

### Sample composition

For all CM activities, participants self-identified in both the racialized and non-racialized categories; some participants did self-identify in more than one category. Of the participants that completed the rating activity (*n* = 72), 41 participants identified as racialized health care users, 23 participants identified as non-racialized health care users, and 11 participants identified as either a racialized or non-racialized health care provider. Of the 41 racialized health care users, 25 participants identified as female and 22 identified as Canadian-born. For this activity, participants self-identified in the following racial/ethnic categories (in alphabetical order): Black, Chinese, Korean, Latin American, South Asian, Southeast Asian, West Asian, White, and other.

### Cluster map

**Fig. 1:**
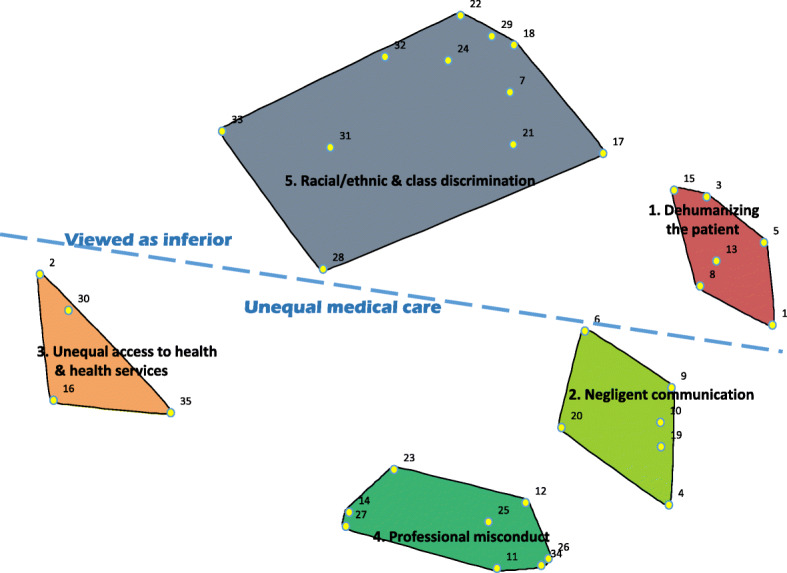
Cluster map with the major conceptual regions of the collective experiences of disrespect or mistreatment in Toronto’s health care system

Figure 1 presents the cluster map and shows the cluster location of each statement. Also, identified from this cluster map are two spatially or conceptually distinct regions. The ‘Viewed as inferior’ conceptual region is located on the right side of the cluster map. This region is dominated by statements of experiences that describe activities or behaviours generally pertaining to interpersonal interactions by health care personnel in which the patient/patient’s family or their needs are viewed as inferior. This region consists of two clusters: ‘Racial/ethnic and class discrimination’ and ‘Dehumanizing the patient’.

The ‘Unequal medical care’ conceptual region is located on the left side of the cluster map. This region is dominated by statements of experiences that describe structural conditions, or activities and behaviours generally pertaining to interpersonal interactions that involve unequal medical access and treatment. This area consists of three clusters: ‘Unequal access to health and health services’, ‘Negligent communication’, and ‘Professional misconduct’.

### Comparison between groups

Table 2 presents the 35 unique statements from the brainstorming activity with the rating for each statement by each of the three groups. A key finding in this study is the different perception of ‘race’/ethnic based discrimination as a reason for the challenges experienced in the health care setting. As compared to racialized and non-racialized health care users, the general perception of health care providers was similar to non-racialized health care users. Specifically, as rated by health care providers and non-racialized health care users, the cluster average was low to moderate suggesting that these groups did *not* perceive ‘race’/ethnic based discrimination as largely contributing to the challenges experienced by patients when receiving health care. Additionally, health care providers rated clusters in the right region of the cluster map higher suggesting that ‘race’/ethnic based discrimination is primarily conceptualized in terms of interpersonal interactions.

**Table 2:** Rating Results for ‘Race’/ethnic Based Discrimination

Cluster	Statement	HCPs	Non-racialized HCUs	Racialized HCUs
Cluster 1: Dehumanizing the patient		moderate	low	moderate
	when the health care provider is disrespectful [15].	high	moderate	high
	when the health care provider belittles or talks down to the patient [3].	moderate	moderate	high
	when the health care provider does not show empathy or sympathy [13].	moderate	low	moderate
	when the health care provider or health care support staff are impatient with the patient [8].	moderate	low	moderate
	when health care provider is impatient with the family after the patient dies [5].	low	low	moderate
	when the health care provider will not listen to the patient or pretends that they do not hear the patient [1].	low	low	moderate
Cluster 2: Negligent communication		low	low	moderate
	when the health care provider does not consider the patient’s concerns about the plan of treatment [20].	moderate	low	moderate
	when the patient’s symptoms are ignored or not taken seriously [10].	moderate	low	moderate
	when the health care provider lies to the patient [19].	low	low	moderate
	when the health care provider does not listen to patient’s medical history before prescribing medication [4].	low	low	moderate
	when the health care support staff places the patient’s phone call on hold and then disconnects them [6].	low	low	low
	when the health care provider willfully misunderstands the patient’s concerns [9].	low	low	moderate
Cluster 3: Unequal access to health & health services		moderate	low	moderate
	when there is little or no access to language interpreters [30].	moderate	moderate	high
	when the health care provider tells the patient that they cannot keep them as their patient because they have enough patients [35].	low	low	low
	when the patient cannot make an appointment to see their health care provider with a two-week timeframe [16].	low	low	low
	when a patient cannot get access to government funded assist programs because of where the patient lives [2].	low	low	moderate
Cluster 4: Professional misconduct		low	low	moderate
	when the patient is discharged prematurely from the hospital [27].	moderate	low	moderate
	when a patient’s message for the health care provider is not relayed by the health care support staff [23].	moderate	low	low
	when the health care provider does not provide the requested information [25].	low	low	low
	when the health care provider does not provide the correct treatment [11].	low	low	low
	when the health care provider does not read the patient’s medical history resulting in negligent care [26].	low	low	low
	when the patient’s pain is not treated [12].	low	low	moderate
	when the health care provider does not provide a referral to see a health care specialist [14].	low	low	moderate
	when the health care provider does not complete a proper assessment [34].	low	low	moderate
Cluster 5: Racial/ethnic & class discrimination		moderate	moderate	high
	when the White male health care provider continuously picks on the non-White patient [22].	high	high	high
	when the patient feels disrespected and not listened to by health care providers because of language issues [31].	high	high	high
	when the health care provider wrongly assumes that the patient does not speak English [32].	high	high	high
	when health care providers or health care staff look down on the patient because of their appearance [18]	moderate	high	high
	when the White health care provider talks to the patient as if they are uneducated [29].	moderate	high	high
	when health care provider is unfamiliar with different religious or cultural practices in caring for a loved one who has died [28].	moderate	high	high
	when the health care provider engages in victim blaming [17].	moderate	moderate	high
	when the patient’s concern is thought of by the health care provider as being superstitious [21].	moderate	moderate	high
	when the patient is wrongly judged to be ‘drug seeking’ [7].	moderate	moderate	moderate
	when a patient on social assistance is treated in a separate area with fewer resources [33].	moderate	low	moderate
	when the patient is looked down on by the health care provider or health care staff for using public transportation [24].	low	low	moderate

*Note.* ^a^ HCPs = health care providers

^b^ HCUs = health care users

### Importance for action/change

**Fig. 2:**
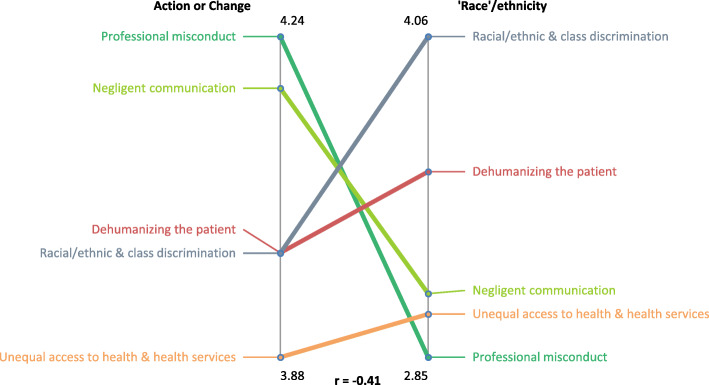
Pattern match comparison between action/change and ‘race’/ethnic based discrimination as rated by racialized health care users in Toronto’s health care system

Another key finding is the prioritization for action/change by racialized health care users. Although racialized health care users rated all clusters moderate to high in terms of importance for action/change and ‘race’/ethnic based discrimination, interestingly, the pattern match graph demonstrates an inverse relationship in the clusters rated high for ‘race’/ethnic based discrimination and for action/change. Figure 2 presents this pattern match graph. The correlation coefficient was r = − 0.41 meaning that there is a moderate inverse relationship between what racialized health care users believe to be most important in terms of ‘race’/ethnic based discrimination as a reason for the challenges experienced in Toronto’s health care system and what they believe to be most important for action/change. Table 3 presents gone-zone statements for all five clusters; these are the statements rated higher for both action/change and ‘race’/ethnic based discrimination as rated by racialized health care users. The ordering of clusters in Table 3 reflect the clusters as ranked by racialized health care users in Fig. 2.

**Table 3:** Go-zone Results on two dimensions: Action/change and ‘Race’/ethnic based discrimination

Cluster	Go-zone statement
Professional misconduct	when the health care provider does not complete a proper assessment [34].
Negligent communication	when the patient’s symptoms are ignored or not taken seriously [10].
	when the health care provider lies to the patient [19].
Dehumanizing the patient	when the health care provider belittles or talks down to the patient [3].
	when the health care provider is disrespectful [15]
Racial/ethnic and class discrimination	when the White male health care provider continuously picks on the non-White patient [22].
	when the White health care provider talks to the patient as if they are uneducated [29].
	when the patient feels disrespected and not listened to by health care providers because of language issues [31].
Unequal access to health and health care	when a patient cannot get access to government funded assist programs because of where the patient lives [2].
	when there is little or no access to language interpreters [30].

*Note.* Clusters are listed in order of ranking priority for action/change as determined by the pattern match data from racialized health care users

## Discussion

Findings from this CM study adds to the literature by providing an understanding of the effect of current health care policies and practices for racial/ethnic groups at the level of the individual in Toronto’s health care system. From the brainstorming activity, participants generated 35 unique statements of disrespect and mistreatment. Of these statements, racialized health care users identified which statements reflected their experience in which they felt discriminated based on their ‘race’/ethnicity. From the sorting and mapping activity, five distinct clusters were identified and thematically labelled by participants: ‘Racial/ethnic and class discrimination’ ’Dehumanizing the patient’ ’Negligent communication’ ’Professional misconduct’ and Unequal access to health and health services. From this cluster map, two spatially or conceptually distinct regions were identified: Viewed as inferior and Unequal medical care. This finding is in keeping with systematic reviews which have demonstrated racial implicit biases by some health care providers [17–20].

From the rating activity, racialized health care users had cluster ratings of moderate to high for the ‘race’/ethnicity rating question this means that racialized health care users reported ‘race’/ethnic based discrimination as largely contributory to the challenges experienced when receiving health care in Toronto’s health care system. In other words, racialized health care users rated ‘race’/ethnic based discrimination as central to the challenges experienced in the health care system.

While our study did not examine the pervasiveness of ‘race’/ethnic based discrimination in Toronto, recent research [68] found pervasive experiences of racial discrimination by health care providers towards Toronto’s Indigenous population. Racial discrimination by health care providers was also positively associated with the unmet health needs of Indigenous health care users. In another recent study which examined the Canadian health care system, several mechanisms of racism which contributed to the unequal access and quality of care of Indigenous health care users were identified. These mechanisms included navigating a system that devalues Indigenous health and wellness, a preference for English and biomedicine, and a lack of consideration for social and economic obstacles to health care accessibility [69].

Behaviours in which racialized patients are viewed as inferior by health care providers are in keeping with the ideology of a racial hierarchy or ideological racism. Ideological racism is the attribution of inferiority and stereotypes to a racial/ethnic group [27, 36]. Racial inferiority is often used for the purpose of justifying racist actions/unequal treatment. This serves to maintain power and privilege for members of the racially dominant group in a racially diverse society [28, 29, 35, 70]. At the micro-level, everyday racism is the process through which this racial hierarchy is maintained; it involves racist practices and behaviours that infiltrate everyday life and are thus, seen as ‘normal’ by the dominant group. Everyday racism is activated by underlying power social relations and thus, adapts to the norms and values of society; at the micro level, the activation of power relations is interconnected to the meso level or institutional level (health care system) [28]. In terms of health care, the ideology of a racial hierarchy has implications for access to resources and quality of care [30, 71]. It affects legislation, policy allocation of resources within institutions, and individual clinician behaviours [72, 73]; it influences medical decisions and interactions, systematically producing institutional racism in health care [74], and a lower quality and access to health care [75]. In our study, reported interpersonal and structural racism in the context of polices intended to support good medical practice and with evidence of racialized health care inequities [1–7] is consistent with the assertion by health equity researchers -- that racialized health inequities are the result of racism at different levels: interpersonal or structural, intentional or unintentional, and perceived or not perceived [35, 70, 76].

In the United States, a landmark report by the Institute of Medicine (IOM) [77], identified that the ‘implicit racial bias and stereotyping’ of racial/ethnic groups by health care providers impacted the treatment of patients in three key areas: unequal treatment/access, lower quality of health care, and under-treatment of pain. Hollingshead and colleagues explain that when ‘race’ is understood as a biological construct, it contributes to the belief held by some health care providers that racial/ethnic groups are less sensitive to pain [78]. This view of ‘race’ as biology continues to impact racialized groups. Studies examining differences in pain management have demonstrated that racialized health care users are under-treated for pain across the lifespan and treatment settings [74, 79, 80]. Evidence has also demonstrated that when guidelines are not well defined, stereotypical inferences based on the patient’s perceived ‘race’/ethnicity contributed to a lower quality of communication during interracial medical interactions [74].

In looking at other countries that examined racism and health care, a recent qualitative study in Europe (Sweden, Germany and Portugal) identified two broad processes of racism and its impact on health care users. The first process was an unequal access to resources leading to silencing and suffering; the second was through inequities in power leading to the erosion of dignity [81]. The authours assert that inequities in health care are concealed as routine everyday practices and interventions, and that deprioritizing the care of racialized groups was rationalized as neutral/objective medical care. In France, using a nationally representative sample, researchers found that immigrants, those of African origin, and of the Muslim religion were more likely to have experienced discrimination in health care settings; this study also found that those that identified as of mixed origin or ‘other religion’ had higher rates of forgoing health care [82]. In New Zealand, a cross-sectional analysis on the experience of racism by health care providers was higher for Maori, Pacific and Asian groups as compared to the European/Other ethnic group; this experience was associated with a higher unmet need and decreased satisfaction with health care [83].

From the pattern match, the clusters rated highest for action/change by racialized health care users were ‘Negligent Communication’ followed by ‘Professional Misconduct’. This finding demonstrates that as compared to the clusters located on the right side of the map, racialized health care users relatively prioritized clusters on left side of the map – ‘Unequal medical care’. In prioritizing unequal access to medical care for taking action/change instead of interpersonal ‘race’/ethnic based discrimination, racialized health care users may be acknowledging the practical reality of staying healthy, for the purpose of returning to work, staying employed, or continuing efforts to access the labour market given that employment income is essential toward meeting the financial challenges of paying for basic necessities such as food, shelter, and medication(s). This ranking may also be due to an awareness by racialized health care users of the denial of institutional racism and by extension, the unwillingness of institutions to develop policies that focus on the improvement of interpersonal behaviours by health care providers. (This data was collected prior to the global movement for racial justice in 2020).

This prioritization by racialized health care users for access to health care aligns with previous research which found that Toronto community members placed greater importance on having accessible social services rather than clinical health services in the communities in which they reside; these services included access to housing, job placement supports and training, and service accessibility [60]. These researchers explain that by participants rating social rather than clinical health services highly, individuals may be acknowledging that while physical wellbeing is important, people also need stable housing, food, and adequate safety in addition to services that can be accessed when and where people need them [60].

According to current research, racialized groups in Toronto continue to be overrepresented in low-income jobs, and members of racialized groups represent 62% of all persons living in poverty [84]. Increasingly, individuals who are unemployed, underemployed, or precariously employed face the financial challenges of paying for prescription medications as outpatient prescriptions are not covered by public funding [85]. Moreover, recent research revealed that in Ontario, racialized workers as compared to non-racialized workers, have lower prescription medication coverage [86]. The prioritization, in this study, by racialized health care users to access medical care may also indicate the need to have multiple health care needs addressed. In Canada, social exclusion from the labour market is linked to poor health [87] with racialized groups at a continued higher risk of labour market social exclusion irrespective of educational attainment [88, 89].

Our study provides a key theoretical contribution; the inverse relationship between ‘race’/ethnic based discrimination and priorities for taking action/change reveals that a broader systems thinking – one that is oriented to the identification and understanding of complex relationships in health care [90] – is essential towards meeting the health needs of racialized health care users. This broader systems thinking within the health care system requires incorporating the social determinants of health and recognizing the importance of improving access to health care for racialized communities.

In concept mapping, a minimum sample size of 10 participants is recommended [58]. To examine the perceptions and opinions specific to our research topic, this study was comprised of 11 health care providers. The finding that health care providers did not report ‘race’/ethnic based discrimination as largely contributing to the experienced challenges of health care users when receiving health care, may be due to the continued use of cultural competence in education and training as the main approach to addressing individual differences in health care. Furthermore, this finding, in the context of cultural competence as the policy of choice for over the past two decades, also sheds light on a broader issue – that nursing, medical, and public health communities seem unwilling to examine institutional racism within medical and public health institutions. Indeed, the literature on racism remains widely unacknowledged in healthcare settings [73, 91]. A recent systematic review also found that the term ‘institutional racism’ was not often explicitly used in public health literature [92].

There is also the continued conceptualization of ‘race’ as a biological construct in the medical curricula. Historically, the understanding of ‘race’ as a biological construct is a remnant of a discredited theory of racial classification developed in the eighteenth century by Johann Blumenbach, a German physician anthropologist, who invoked the false idea that races are biological and that there are ranked subdivisions of the human species with ‘Caucasians’ (White) placed as the superior race or at the top of the racial classification or hierarchy [93–95]. Indeed, there are renewed calls for medical schools to stop promoting ‘race’ as biology and offering specific recommendations for improvements [96] including an anti-racist approach [97].

Contributing to racial/ethnic stereotypes within health care is the biological essentialism seen with the pharmacogenetic research development and marketing of medications that target specific racialized groups. For example, BiDil, a cardiovascular medication, was the first medication approved by the American Federal Drug Administration that specifically targeted the African American population. This contemporary ‘race’ based research has been viewed as deeply flawed [70, 98–101]. Therefore, while there are new interventions targeted towards addressing implicit racial bias at the level of the individual [102, 103], these interventions fail to address institutional racism. Importantly, as asserted by Essed [28], we cannot place the individual outside of the institution as this would only serve to sever the rules and regulations from the people who enact them. Therefore, given that racism is fundamentally structural and systemic, to achieve individual change, the entry point for policy must begin at the level of the institution – with institutional policies.

In this study, racialized health care users reported that access to and quality of medical care are challenges currently experienced in Toronto’s health care system. Since the ideology of racial inferiority creates an environment whereby the macro societal system of racism is the foundation for both meso institutional and micro individual-level discrimination, to reduce institutional racism, political will is needed to eliminate the ideology of racial inferiority that is currently pervasive in society [104]. Accordingly, policy changes are essential across multiple domains of health care (e.g. primary public health care, walk-in clinics, and tertiary care such as hospitals) and other social institutions [104]; specifically, anti-racism policies are needed.

Anti-racism is a theoretically informed political praxis and is needed to effectively challenge and *overcome* racism [105] starting with explicitly naming the issue of racism and social differences of power and equity instead of culture [106, 107]. Anti-racism policies would explicitly identify structured unequal power relation systems of oppression and domination in order to explain the complex processes that generate racism, the continuation of racism, and its impact [108]. An anti-racist framework that focuses on power and equity is needed to confront the myth of neutrality by understanding and connecting racism to the policy realm and social institutions in order to explain how racism is reproduced and its impact for racialized groups [30, 32, 108, 109]. This theoretically informed approach is also needed to explicitly name racism as a form of oppression [110] and to hold institutions accountable [111].Thus, in terms of implications, to meet the health care needs of racialized health care users in terms of access and quality of medical care, health care providers must begin by acknowledging racism [76]. To initiate widespread change within regulatory and educational social institutions, the medical and nursing leadership in Canada and Ontario must advocate for anti-racism position statements, practice guidelines, and educational curriculum.

Our findings demonstrated that health care providers primarily conceptualized ‘race’/ethnic based discrimination in terms of *interpersonal* interactions by health care personnel in which the patient/patient’s family or their needs are viewed as inferior. However, an understanding of racism as a psychological phenomenon limits an understanding of racism as an unequal integrated system of policies and laws that has political consequences in terms of allocations of resources [112]. Additionally, in this study, health care providers did *not* perceive ‘race’/ethnic based discrimination as central to the challenges experienced by health care users in Toronto’s health care system. Taken together, these findings underscore that the current reliance on a cultural competence policy in the health care setting ignores the existence of institutional racism in health care and in other areas at the meso level of society including the labour market, and thus the socioeconomic hardships faced by racialized communities.

Medical education continues in teaching ‘race’ primarily as a biological construct [46] and as a result the health care system continues to be structured around a biomedical model of health care delivery. To avoid crude biological essentializations, stereotypical generalizations, and the continued pathologization of racialized groups who are experiencing racism, there is a foundational requirement to explicitly conceptualize ‘race’/ethnicity as a social construct in medical education curricula – as a set of historically specific political, economic, and ideological processes. The current liberal notion in the health care setting of ‘treating everyone the same’ when it comes to understanding the needs of racialized health care users fails to acknowledge these historical processes and the significant impact of racism on health and health care needs [108].

The education of health professionals must include anti-racist training based on an explicit anti-racist pedagogy. An anti-racist education would replace the current cultural competence approach which currently serves to obscure broader institutional and societal influences on health and health care. An anti-racist pedagogy is theoretically grounded in a critical pedagogy and orients learners through an analysis of systems of power and domination ‘to explain and counteract the persistence of racism using praxis (theory and practice) as its focus to promote social justice’ ([113], p. 3), and by taking a broad contextual and structural understanding of racism [113–115].

Health care organizations must develop a hospital anti-racism task force that includes all levels of staff (including managers, administrative, and front line) to begin ongoing dialogs about racism issues with both racialized and non-racialized health care employees/personnel within the organization. An anti-racism strategy would also include incorporating anti-racist work at the executive levels of management and board of directors through discussion on the impact of racism on both patients and the organization. Managerial implications include developing policies that respond effectively to the relationships of oppression and privilege in the areas of hiring, promoting, and professional development. The development of policies, relational practice, and a work environment that promotes inclusiveness and addresses racism is required. More broadly, they need to develop policies that are responsive and accountable to the communities they serve [116] and in particular, to racialized health care users.

Practical implications for health care providers using an anti-racist approach includes the need to focus on minimizing power imbalances between health care providers and racialized health care users. Towards this aim, health care providers should tailor health care for racialized health care users by focusing on the patient’s structural determinants or socio-economic context and their social position, and to prioritize care as determined by the patient’s view of their needs [117, 118].

This study has several limitations. One key limitation is that all CM activities were conducted in English. Consequently, there is an absence of experiences specific to non-English speaking racialized health care users (e.g. non-economic immigrants such as refugees and family class sponsored immigrants). Another limitation is that this study was completed within a limited time frame which hindered the recruitment of additional health care providers and thus, an exploration of differences within this group (e.g. by ‘race’/ethnicity).

A strength of this study is the stress value. For this CM study, the stress value is 0.18 demonstrating a good fit, thus supporting the validity of the conceptual model or cluster map. In other words, when sorting, all participants generally agreed upon the grouping of statements or conceptual phenomenon, and by extension, the regions on the cluster map in which the clusters are located.

This study’s approach to enquiry is consistent with a CM participatory approach [57, 59]. Members of the Toronto and GTA communities participated in several phases of the research process including the generation and interpretation of data. Finally and importantly, our study’s design and recommendations are consistent a with CM approach that calls for priority agenda setting for health and health care as determined by the communities negatively impacted from policies and practices [119].

The above findings have implications for future research examining the impact of current policies and practices in the health care setting. We used purposive sampling in this semi-qualitative study to identify a wide variety of mechanisms contributing to everyday racism in the health care system. Future quantitative studies could examine the prevalence or pervasiveness of everyday racism in the health care system. Using a larger sample size, future studies could also examine the differences in the understanding of ‘race’/ethnic based discrimination between racialized and non-racialized health care providers, and between different types of health care providers. Future research, including interpreters and translated materials, is needed to identify mechanisms of ‘race’/ethnic based discrimination for racialized health care users who are limited in or do not speak English.

## Conclusion

In summary, our findings identify how racialized health care users experience everyday racism when receiving health care and this is important to consider in the development of future research and interventions aimed at addressing institutional racism in the health care setting. Racialized health care users from Toronto (Canada’s largest city) and the Greater Toronto Area, reported ‘race’/ethnic based discrimination as largely contributory to the challenges experienced when receiving health care. Racialized health care users also prioritized unequal access to medical care for taking action/change. To support the elimination of institutional racism, anti-racist policies are needed to explicitly name the issue of racism and to address the centrality of unequal power social relations and everyday racism in the health care system.

## Data Availability

The data for this study are not publicly available.

## References

[1] Public Health Agency of Canada (2018). Key health inequalities in Canada: a national portrait. Pan-Canadian Health Inequalities Reporting Initiative.

[2] Ramraj C, Shahidi FV, Darity W, Kawachi I, Zuberi D, Siddiqi A (2016). Equally inequitable? A cross-national comparative study of racial health inequalities in the United States and Canada. Soc Sci Med.

[3] Siddiqi A, Shahidi FV, Ramraj C, Williams DR (2017). Associations between race, discrimination and risk for chronic disease in a population-based sample from Canada. Soc Sci Med.

[4] Siddiqi AA, Wang S, Quinn K, Nguyen QC, Christy AD (2016). Racial disparities in access to care under conditions of universal coverage. Am J Prev Med.

[5] Veenstra G, Patterson AC (2016). Black–white health inequalities in Canada. J Immigr Minor Health.

[6] Veenstra G, Patterson AC (2016). South Asian-white health inequalities in Canada: intersections with gender and immigrant status. Ethn Health.

[7] Toronto Public Health (2013). Racialization and health inequities in Toronto.

[8] McKenzie K (2020). Toronto and Peel have reported race-based and socio-demographic data – now we need action. Wellesley Institute.

[9] Allen K. This Black PSW with COVID-19 was sent home from hospital Two days later he died. Toronto Star. Weblog. [Online] Available from: https://www.thestar.com/news/gta/2020/05/23/this-black-psw-with-covid-19-was-sent-home-from-hospital-two-days-later-he-died.html [Accessed 23 May 2020].

[10] Marmot M (2015). The health gap: the challenge of an unequal world.

[11] Schrecker T, Bambra C (2015). How politics makes us sick: neoliberal epidemics.

[12] Smith KE, Bambra C, Hill SE (2016). Health inequities: critical perspectives.

[13] Commission on the Social Determinants of Health. Closing the gap in generation: Health equity through action on the Social Determinants of Health*.* World Health Organization. http://whqlibdoc.who.int/publications/2008/9789241563703_eng.pdf (2008). Accessed 13 Nov 2020.

[14] Hyman I, Wray R (2013). Health inequalities and racialized groups: a review of the evidence.

[15] Nestel S (2012). Colour coded health care: the impact of race and racism on Canadians’ health. Wellesley Institute.

[16] Khan M, Kobayashi K, Lee SM, Vang Z (2015). Visible minorities in Canadian health data and research.

[17] Dehon E, Weiss N, Jones J, Faulconer W, Hinton E, Sterling S (2017). A systematic review of the impact of physician implicit racial bias on clinical decision making. Acad Emerg Med.

[18] FitzGerald C, Hurst S (2017). Implicit bias in healthcare professionals: a systematic review. BMC Med Ethics.

[19] Hall WJ, Chapman MV, Lee KM, Merino YM, Thomas TW, Payne BK, Eng E, Day SH, Coyne-Beasley T (2015). Implicit racial/ethnic bias among health care professionals and its influence on health care outcomes: a systematic review. Am J Public Health Res..

[20] Paradies Y, Truong M, Priest N (2014). A systematic review of the extent and measurement of healthcare provider racism. J Gen Intern Med.

[21] Benjamins MR, Whitman S (2014). Relationships between discrimination in health care and health care outcomes among four race/ethnic groups. J Behav Med.

[22] Ben J, Cormack D, Harris R, Paradies Y (2017). Racism and health service utilisation: a systematic review and meta-analysis. PLoS One.

[23] Mouzon DM, Taylor RJ, Woodward AT, Chatters LM (2017). Everyday racial discrimination, everyday non-racial discrimination, and physical health among African-Americans. J Ethn Cult Divers Soc Work.

[24] Mawani F, Ansara D, Smylie J, Forte T, Mahabir DF, Hyman I, Siddiqi A, McKenzie K, O'Campo P (2019). Everyday discrimination and physical and mental health in Canada: an examination of effect modification by racialized/Indigenous identity, gender, and income. Manuscript in preparation.

[25] Muntaner C (1999). Invited commentary: social mechanisms, race, and social epidemiology. Am J Epidemiol.

[26] Muntaner C (2013). Invited commentary: on the future of social epidemiology—a case for scientific realism. Am J Epidemiol.

[27] Muntaner C, Nieto FJ, O'Campo P (1996). The Bell curve: on race, social class, and epidemiologic research. Am J Epidemiol.

[28] Essed P (1991). Understanding everyday racism: an interdisciplinary theory.

[29] Jones CP (2000). Levels of racism: a theoretic framework and a gardener’s tale. Am J Public Health Res..

[30] Feagin J, Bennefield Z (2014). Systemic racism and US health care. Soc Sci Med.

[31] Krieger N (2016). Living and dying at the crossroads: racism, embodiment, and why theory is essential for a public health of consequence. Am J Public Health Res.

[32] Williams DR, Rucker TD (2000). Understanding and addressing racial disparities in health care. Health Care Financ Rev.

[33] Bradby H (2010). What do we mean by ‘racism’? Conceptualising the range of what we call racism in health care settings: A commentary on peek et al. Soc Sci Med.

[34] Bradby H (2012). Race, ethnicity and health: the costs and benefits of conceptualising racism and ethnicity. Soc Sci Med.

[35] Krieger N (2014). Discrimination and health inequities. Int J Health Serv.

[36] Williams DR, Lawrence JA, Davis BA (2019). Racism and health: evidence and needed research. Annu Rev Public Health.

[37] Canada Health Act. Justice Laws Website. 1985. http://laws-lois.justice.gc.ca/PDF/C-6.pdf. Accessed 12 Nov 2020.

[38] Hallstrom LK, Raphael D (2016). Public policy, equality, and health in Canada. Social determinants of health: Canadian perspectives.

[39] Esping-Andersen G (1990). The three worlds of welfare capitalism.

[40] Ruckert A, Caldbick S, Labonté R (2015). Equity in Times of Austerity: Ontario’s Revenue Crisis in Historical Perspective. Can Rev Social Policy.

[41] Bryant T (2016). Health policy in broader perspective: welfare states and public policy. Health policy in Canada.

[42] Silver J (2014). About Canada: poverty.

[43] Gaffney A, Muntaner C, Waitzkin H, the Working Group on Health Beyond Capitalism (2018). Austerity and health care. Health care under the knife: moving beyond capitalism for our health.

[44] Boyd RW, Lindo EG, Weeks LD, McLemore MR (2020). On racism: a new standard for publishing on racial health inequities. Health Affairs Blog.

[45] Nuru-Jeter AM, Michaels EK, Thomas MD, Reeves AN, Thorpe RJ, LaVeist TA (2018). Relative roles of race versus socioeconomic position in studies of health inequalities: a matter of interpretation. Annu Rev Public Health.

[46] Sharma M, Kuper A (2017). The elephant in the room: talking race in medical education. Adv Health Sci Educ.

[47] Williams DR (1997). Race and health: basic questions, emerging directions. Ann Epidemiol.

[48] Ahmad WIU, Bradby H (2007). Locating ethnicity and health: exploring concepts and contexts. Sociol Health Illn.

[49] Muntaner C, Nagoshi C, Diala C (2001). Racial ideology and explanations for health inequalities among middle-class whites. Int J Health Serv.

[50] Anderson LM, Scrimshaw SC, Fullilove MT, Fielding JE, Normand J (2003). Task force on community preventive services. Culturally competent healthcare systems: a systematic review. Am J Prev Med.

[51] Beach MC, Price EG, Gary TL, Robinson KA, Gozu A, Palacio A, Powe NR (2005). Cultural competency: a systematic review of health care provider educational interventions. Med Care.

[52] Gallagher RW, Polanin JR (2015). A meta-analysis of educational interventions designed to enhance cultural competence in professional nurses and nursing students. Nurse Educ Today.

[53] Truong M, Paradies Y, Priest N (2014). Interventions to improve cultural competency in healthcare: a systematic review of reviews. BMC Health Serv Res.

[54] Alleyne J, Papadopoulos I, Tilki M (1994). Antiracism within transcultural nurse education. Br J Nurs.

[55] Shavers VL, Fagan P, Jones D, Klein WM, Boyington J, Moten C, Rorie E (2012). The state of research on racial/ethnic discrimination in the receipt of health care. Am J Public Health.

[56] Trochim WM (1989). An introduction to concept mapping for planning and evaluation. Eval Program Plann..

[57] Burke JG, O’Campo P, Peak GL, Gielen AC, McDonnell KA, Trochim WM (2005). An introduction to concept mapping as a participatory public health research method. Qual Health Res.

[58] Kane M, Trochim W (2007). Concept mapping for planning and evaluation.

[59] O’Campo P, Burke J, Peak GL, McDonnell KA, Gielen AC (2005). Uncovering neighbourhood influences on intimate partner violence using concept mapping. J Epidemiol Community Health.

[60] Velonis AJ, Molnar A, Lee-Foon N, Rahim A, Boushel M, O’Campo P (2018). “One program that could improve health in this neighbourhood is _?” using concept mapping to engage communities as part of a health and human services needs assessment. BMC Health Serv Res.

[61] Lobb R, Pinto AD, Lofters A (2013). Using concept mapping in the knowledge-to-action process to compare stakeholder opinions on barriers to use of cancer screening among south Asians. Implement Sci Commun.

[62] The Concept System® Global MAX™ (Build 2018.187.12) [Web-based Platform]. Ithaca, NY. http://www.conceptsystemsglobal.com (2018). Accessed Oct 4 2018.

[63] Anderberg MR (1988). Cluster analysis for applications.

[64] Everitt B (1980). Cluster analysis.

[65] Moghaddam FM, Studer C (1997). The sky is falling, but not on me: a cautionary tale of illusions of control, in four acts. Cross Cult Res.

[66] Lewis TT, Cogburn CD, Williams DR (2015). Self-reported experiences of discrimination and health: scientific advances, ongoing controversies, and emerging issues. Annu Rev Clin Psychol.

[67] O’Campo P (2014). Concept mapping: a short introduction.

[68] Kitching GT, Firestone M, Schei B, Wolfe S, Bourgeois C, O’Campo P, Rotondi M, Nisenbaum R, Maddox R, Smylie J (2020). Unmet health needs and discrimination by healthcare providers among an indigenous population in Toronto, Canada. Can J Public Health.

[69] Phillips-Beck W, Eni R, Lavoie JG, Avery Kinew K, Kyoon Achan G, Katz A (2020). Confronting racism within the Canadian healthcare system: systemic exclusion of first nations from quality and consistent care. Int J Environ Res Public Health.

[70] Williams DR, Mohammed SA (2009). Discrimination and racial disparities in health: evidence and needed research. J Behav Med.

[71] Rambachan A (2018). Overcoming the racial hierarchy: the history and medical consequences of “caucasian”. J Racial Ethn Health Disparities.

[72] Fiscella K, Sanders MR (2016). Racial and ethnic disparities in the quality of health care. Annu Rev Public Health.

[73] Williams DR, Wyatt R (2015). Racial bias in health care and health: challenges and opportunities. JAMA Netw Open.

[74] Dovidio JF, Fiske ST (2012). Under the radar: how unexamined biases in decision-making processes in clinical interactions can contribute to health care disparities. Am J Public Health.

[75] Brewer LC, Cooper LA (2014). State of the art in science: race, discrimination, and cardiovascular disease. Virtual Mentor.

[76] Jones CP. Confronting institutionalized racism. Phylon. 2002;50:7–22.

[77] Smedley BD, Stith AY, Nelson AR (2003). Unequal treatment: confronting racial and ethnic disparities in health care.

[78] Hollingshead NA, Meints SM, Miller MM, Robinson ME, Hirsh AT (2016). A comparison of race-related pain stereotypes held by white and Black individuals. J Appl Soc Psychol.

[79] Anderson KO, Green CR, Payne R (2009). Racial and ethnic disparities in pain: causes and consequences of unequal care. J Pain.

[80] Green CR, Anderson KO, Baker TA, Campbell LC, Decker S, Fillingim RB, Kaloukalani DA, Lasch KE, Myers C, Tait RC, Todd KH (2003). The unequal burden of pain: confronting racial and ethnic disparities in pain. Pain Med.

[81] Hamed S, Thapar-Björkert S, Bradby H, Ahlberg BM (2020). Racism in European health care: structural violence and beyond. Qual Health Res.

[82] Rivenbark JG, Ichou M (2020). Discrimination in healthcare as a barrier to care: experiences of socially disadvantaged populations in France from a nationally representative survey. BMC Public Health.

[83] Harris RB, Cormack DM, Stanley J (2019). Experience of racism and associations with unmet need and healthcare satisfaction: the 2011/12 adult New Zealand health survey. Aust N Z J Public Health.

[84] Colour of Poverty. Racialized poverty in income and social assistance. https://colourofpovertyca.files.wordpress.com/2019/03/cop-coc-fact-sheet-6-racialized-poverty-in-income-social-assistance-1.pdf (2019). Accessed 13 Nov 2020.

[85] Chowdhury MZ, Chowdhury MA (2018). Canadian health care system: who should pay for all medically beneficial treatments? A burning issue. Int J Health Serv.

[86] Cheff R, Hill M, Iveniuk J (2019). Who benefits? Gaps in medication coverage for Ontario workers. Wellesley Institute.

[87] Labonté R, Cobbett E, Orsini M, Spitzer D, Schrecker T, Ruckert A (2015). Globalization and the health of Canadians: ‘having a job is the most important thing’. Glob Health.

[88] Galabuzi G (2006). The economic exclusion of racialized communities: a statistical profile. Canada’s economic apartheid: the social exclusion of racialized groups in the new century.

[89] Galabuzi G, Raphael D (2016). Social Exclusion. Social determinants of health: Canadian perspectives.

[90] Leischow SJ, Best A, Trochim WM, Clark PI, Gallagher RS, Marcus SE, Matthews E (2008). Systems thinking to improve the public's health. Am J Prev Med.

[91] Thorne S (2017). Isn’t it high time we talked openly about racism?. Nurs Inq.

[92] Hardeman RR, Murphy KA, Karbeah JM, Kozhimannil KB (2018). Naming institutionalized racism in the public health literature: a systematic literature review. Public Health Rep.

[93] Marshall G, Harding S (1993). Racial Classifications. The “racial” economy of science: toward a democratic future. Indiana University Press.

[94] Mukhopadhyay CC, Pollock M (2008). Getting rid of the word “caucasian”. Everyday anti-racism.

[95] Painter NI (2010). The history of white people.

[96] Amutah C, Greenidge K, Mante A, Munyikwa M, Surya SL, Higginbotham E, Jones DS, Lavizzo-Mourey R, Roberts D, Tsai J, Aysola J. Misrepresenting race—the role of medical schools in propagating physician bias. N Engl J Med. 2021;1–7. 10.1056/nejmms2025768. 10.1056/NEJMms2025768 33406326

[97] Yousif H, Ayogu N, Bell T. The path forward—an antiracist approach to academic medicine. N Engl J Med. 2020;1–2. 10.1056/NEJMpv2024535. 10.1056/NEJMpv2024535 32961043

[98] Garrod JZ (2006). A brave old world: an analysis of scientific racism and BiDil®. Mcgill J Med.

[99] Brody H, Hunt LM (2006). BiDil: assessing a race-based pharmaceutical. Ann Fam Med.

[100] Cooper RS, Kaufman JS (2003). Race and genomics. N Engl J Med medicine.

[101] Williams DR, Mohammed SA, Leavell J, Collins C (2010). Race, socioeconomic status and health: complexities, ongoing challenges and research opportunities. Ann N Y Acad Sci.

[102] Burgess D, Van Ryn M, Dovidio J, Saha S (2007). Reducing racial bias among health care providers: lessons from social-cognitive psychology. J Gen Intern Med.

[103] Devine PG, Forscher PS, Austin AJ, Cox WT (2012). Long-term reduction in implicit race bias: a prejudice habit-breaking intervention. J Exp Soc Psychol.

[104] Williams DR, Cooper LA (2019). Reducing racial inequities in health: using what we already know to take action. Int J Environ Res Public Health.

[105] Bakan AB, Dua E, Bakan AB, Dua E (2014). Introducing the questions, reframing the dialogue. Theorizing anti-racism: linkages in Marxism and critical race theories.

[106] Dei GJS, Dei GJS, Calliste A (2000). Towards an anti-racism discursive framework. Power, knowledge and anti-racism education.

[107] Este D, Lorenzetti L, Sato C, Este D, Lorenzetti L, Sato C (2018). The colourblind society. Racism and anti-racism in Canada.

[108] McGibbon EA, Etowa JB. Anti-racist health care practice. Toronto: Canadian Scholars’ Press; 2009.

[109] Krieger N, Rowley DL, Herman AA, Avery B, Phillips MT (1993). Racism, sexism, and social class: implications for studies of health, disease, and well-being. Am J Prev Med.

[110] Bakan AB, Bakan AB, Dua E (2014). Marxism and anti-racism: rethinking the politics of difference. Theorizing anti-racism: linkages in Marxism and critical race theories.

[111] Deckers CM, Dei GS, McDermott M (2014). Moving toward and anti-racism curriculum. Politics of anti-racism education: in search of stratigies for transformative learning.

[112] Bonilla-Silva E (1997). Rethinking racism: toward a structural interpretation. Am Sociol Rev.

[113] Garneau AB, Browne AJ, Varcoe C (2018). Drawing on antiracist approaches toward a critical antidiscriminatory pedagogy for nursing. Nurs Inq.

[114] McGibbon E (2019). Truth and reconciliation: healthcare organizational leadership. Healthc Manage Forum.

[115] Metzl JM, Petty J, Olowojoba OV (2018). Using a structural competency framework to teach structural racism in pre-health education. Soc Sci Med.

[116] Greene MP, Levine MP. Promoting organization and systematic change. In: Carten A, Siskind A, Greene MP, editors. Strategies for deconstructing racism in the health and human services. New York: Oxford University Press; 2016. p. 3–17.

[117] Corneau S, Stergiopoulos V (2012). More than being against it: anti-racism and anti-oppression in mental health services. Transcult Psychiatry.

[118] Goel R, Buchman S, Meili R, Woollard R (2016). Social accountability at the micro level: one patient at a time. Can Fam Physician.

[119] Anderson LA, Slonim A (2017). Perspectives on the strategic uses of concept mapping to address public health challenges. Eval Program Plann.

